# A public health perspective of aging: do hyper-inflammatory syndromes such as COVID-19, SARS, ARDS, cytokine storm syndrome, and post-ICU syndrome accelerate short- and long-term inflammaging?

**DOI:** 10.1186/s12979-020-00196-8

**Published:** 2020-08-24

**Authors:** Arsun Bektas, Shepherd H. Schurman, Claudio Franceschi, Luigi Ferrucci

**Affiliations:** 1 Translational Gerontology Branch, National Institute on Aging, National Institutes of Health, 251 Bayview Blvd, Baltimore, MD 21224 USA; 2 Clinical Research Branch, National Institute of Environmental Health Sciences, National Institutes of Health, 111 TW Alexander Dr, Research Triangle Park, NC 27709 USA; 3 Alma Mater Studiorum University of Bologna, Bologna, Italy; 4 Laboratory of Systems Biology of Healthy Aging and Department of Applied Mathematics, Lobachevsky State University, Nizhny Novgorod, Russia

**Keywords:** Inflammaging, COVID-19, Inflammation, Cytokine storm syndrome

## Abstract

A central clinical question as the world deals with the COVID-19 pandemic is what the long-term sequelae for the millions of individuals will be who recover from the hyperinflammatory state characterizing COVID-19 and in particular for the hundreds of thousands who are ill enough to need hospitalization and in particular ICU care. Even when the pandemic is finally controlled, will COVID-19 survivors face exaggerated internal inflammatory processes, worsening co-morbidities, and increased susceptibility to age-related diseases? Clues for what may happen in post-COVID-19 patients can be elicited from those who recovered from other conditions that lead to similar hyperinflammatory states such as Severe Acute Respiratory Syndrome (SARS), acute respiratory disease syndrome (ARDS), cytokine storm syndrome, and post-ICU syndrome. The short-and long-term sequalae following recovery from each of these conditions suggests that these syndromes lead to an accelerated state of chronic subclinical systemic inflammation often seen in aging (termed inflammaging) resulting in increased and worsening age-related conditions including frailty even in younger individuals.

## Background

COVID-19, a mild to severe respiratory syndrome that follows infection with Severe Acute Respiratory Syndrome Coronavirus 2 (SARS-CoV-2), was first identified in Wuhan, China in December 2019 and rapidly became a pandemic that affected millions of people, causing substantial mortality around the world. Most individuals infected with SARS-CoV-2 develop a flu-like, mild clinical syndrome (non-pneumonia or a mild atypical pneumonia), and a sizeable proportion (~ 20%) require hospitalization (dyspnea, low oxygen saturation), with a substantial proportion leading to a critical illness (respiratory failure, septic shock, and/or multiple organ dysfunction) and death [1, 2].

While the mechanism by which only some individuals develop severe respiratory pathology is still not fully clarified, a number of studies have shown that patients who are ill enough to require hospitalization tend to be older persons affected by multimorbidity, including hypertension, diabetes, and/or obesity [3]. Disease severity in hospitalized patients is characterized by severe pneumonia associated with overt inflammatory reaction characterized by high C-reactive protein (CRP) and interleukin-6 (IL-6), low albumin, high sedimentation rate, low eosinophils, and lymphopenia. Hospitalized individuals also have increased lactate dehydrogenase (LDH), a marker of cellular death, often associated with altered coagulation [4, 5]. High LDH, low lymphocyte count and high levels of high-sensitivity CRP predict mortality of individual patients more than 10 days in advance with greater than 90% accuracy [6]. Several meta-analyses also associated IL-6 levels with the severity of COVID-19 syndrome [7–9].

Thus, an extraordinary proliferation of studies published over the last few months suggests that SARS-CoV-2 infection unleashes a powerful, and apparently uncontrolled inflammatory response that most likely adds to the tissue damage already caused by the viral infection toward the COVID-19 underlying pathology. The high concentration of pro-inflammatory mediators, which has been named ‘the cytokine storm,’ damages the cardiac, hepatic, and renal systems leading to tumor necrosis factor (TNF)-mediated multi-organ system failure and/or death [10]. SARS-CoV-2 mostly affects levels of pro-inflammatory cytokines/chemokines typical of T helper 1 (TH1) cell response such as IL-6, IFNγ, IP-10, and MCP1 [11, 12], that attract monocytes and T cells to the infected site [13, 14], which probably contributes to the lymphopenia and increased neutrophil–lymphocyte ratio seen in ~ 80% of patients [15, 16]. Noteworthy, while severe COVID-19 affects disproportionally older people, systemic inflammation from SARS-CoV-2 is detected in patients of all age groups, including a severe multisystem inflammatory syndrome with features of Kawasaki disease recently identified in children [17, 18].

## COVID-19 and inflammaging

**Fig. 1:**
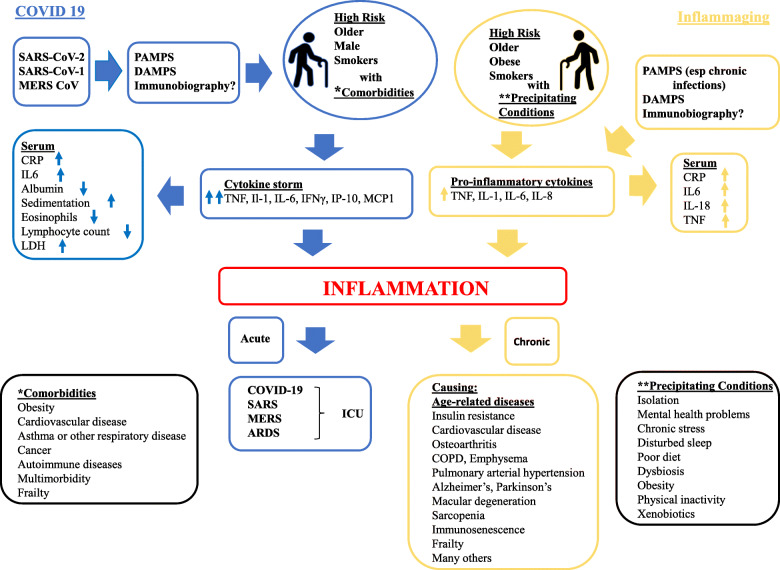
COVID-19 and Inflammaging. *Inflammaging (highlighted in yellow)*. Systemic, sustained chronic inflammation due to persistent tissue damage, environmental stressors, unhealthy lifestyle, and social and psychological stress is associated with the risk of developing many chronic diseases. Older, obese, and/or smokers with precipitating conditions, are at higher risk. Exogenous compounds such as bacteria or viral fragments including elements of microbiota (pathogen-associated molecular patterns, or PAMPs) or endogenously produced chemical compounds that structurally mimic exogenous compounds (damage-associated molecular patterns or DAMPs) interact with sensors, expressed on the cell surface and in the cytoplasm, to trigger inflammatory responses and pro-inflammatory cytokine secretion (eg. tumor necrosis factor [TNF], interleukin-1 [IL-1], IL-6, and IL-8, and type 1 interferon) that contribute to the state of chronic subclinical systemic inflammation seen in aging (termed inflammaging). Immunobiography, the individual’s history of exposure to certain microorganisms (eg. HIV and CMV) or antigens, may condition the degree and characteristic of the inflammatory response to various stimuli. Inflammaging, which results in an increased incidence and worsening of age-related conditions, is characterized clinically by higher levels of several inflammatory blood biomarkers, including C-reactive protein (CRP), IL-6, IL-18, and TNF. *COVID-19 (highlighted in blue)*. SARS-CoV-2 infection, like SARS-CoV-1 and MERS CoV, can unleash a powerful, and apparently uncontrolled acute inflammatory response in individuals who may be more susceptible because of biological differences in their response to PAMPS, DAMPS, and factors related to immunobiography. Interestingly, prognostic factors for mortality from COVID-19 are similar to those that have been found associated with a high likelihood and prospective risk of chronic inflammation, namely older age, male sex, obesity, smoking, cardiovascular diseases, asthma or other respiratory disease, cancer, autoimmune diseases, multimorbidity, and frailty. This similarity may suggest that the same mechanisms underlying inflammaging are also those that modulate the clinical course of COVID-19 across a wide range of severity, from a mild flu-like syndrome to a severe respiratory failure with high mortality risk. In those with more severe disease, high concentrations of pro-inflammatory mediators, which has been named ‘the cytokine storm,’ affects levels of pro-inflammatory cytokines/chemokines typical of T helper 1 cell response such as IL-6, IFNγ, IP-10, and MCP1. COVID-19 disease severity in hospitalized patients is characterized by severe pneumonia associated with an overt inflammatory reaction typified by high plasma CRP and IL-6, low albumin, high sedimentation rate, low eosinophils, and lymphopenia, often leading to intensive care treatment

Inflammation is a physiological response essential to oppose infections and contribute to tissue repair. Systemic, sustained chronic inflammation due to persistent tissue damage, environmental stressors, unhealthy lifestyle, and social and psychological stress is associated with the risk of developing many chronic diseases (Fig. 1) including metabolic syndrome, type 2 diabetes, non-alcoholic fatty liver disease, cardiovascular disease (CVD), sarcopenia, osteoporosis, immunosenescence, autoimmune disorders, cancer, chronic kidney disease, neurodegenerative disorders, depression, and possibly accelerated cognitive decline and dementia [19]. The mechanisms by which cellular damage and dysfunction cause ailments such as atherosclerosis, chronic ischemia, central obesity or degenerative diseases have been described and are relatively clear. However, in older individuals these “direct” mechanisms only explain a relatively small portion of the pro-inflammatory state that is observed progressively more frequently as people age. Other conditions associated with a chronic inflammatory response are more “complex” and still not well understood. Mechanisms include isolation, mental health problems, chronic stress, disturbed sleep, poor diet, dysbiosis, obesity, physical inactivity, and xenobiotics [19]. One of the stress-response mechanisms recently implicated in the pathogenesis of sustained chronic inflammation is the accumulation of cellular senescence. Cells become senescent in response to many stresses, including genomic instability, oxidative stress, energetic crisis, metabolic derangement, altered proteostasis, and many others. The hallmarks of cellular senescence are an irreversible arrest in cell proliferative capacity, resistance to apoptosis and the massive secretion of senescence-associated secretory phenotype (SASP) molecules that include cytokines, chemokines and chemokines among other molecular species. Research in animal models and some initial data in humans suggest the hypothesis that the typical pro-inflammatory state of aging is in large part sustained by the accumulation of senescent cells and the spilling in the blood of SASP pro-inflammatory factors. This hypothesis may explain why chronic inflammation has been associated with many age-related chronic conditions such as insulin resistance, CVD, osteoarthritis, chronic obstructive pulmonary disease (COPD), emphysema, pulmonary arterial hypertension, Alzheimer’s, Parkinson’s, and macular degeneration, which are all characterized by the accumulation of senescent cells [19–23]. Importantly, there is strong evidence from model organisms, and more recently from human studies, that dysfunction in many of the mechanisms of aging biology, the so-called hallmarks of aging, all converge into a pro-inflammatory response. Relevant for the discussion here, exogenous compounds such as bacteria or viral fragments including elements of microbiota (pathogen-associated molecular patterns, or PAMPs) or endogenously produced chemical compounds that structurally mimic exogenous compounds, such as uric acid crystals or mitochondrial fragments (damage-associated molecular patterns or DAMPs) interact with sensors, referred to as pattern recognition receptors (PPRs), expressed on the cell surface and in the cytoplasm (e.g. Toll-like receptor, NOD-like receptor, cyclic GMP-AMP synthase, aryl hydrocarbon receptor) to trigger inflammatory responses (e.g. IL-1β, IL-18, NF-κB activation, pro-inflammatory cytokine secretion [e.g. TNF, IL-1, IL-6, and IL-8], and type 1 interferon) that contribute to the state of chronic subclinical systemic inflammation seen in aging (termed inflammaging) [24]. Indeed, inflammaging, which results in  an increased incidence and worsening of age-related conditions [25–27], is characterized clinically by higher levels of several inflammatory blood biomarkers, including CRP, IL-6, IL-18, and TNF [28]. IL-6 serum levels also predict incident disability and frailty [29], with high IL-6 scores being related to lower walking speeds [30], and the risk of developing mobility disability increasing linearly for IL-6 levels higher than 2.5 pg/ml [31].

The array of mechanisms outlined above may all be possible explanations of how a bacterial or viral infection may trigger an inflammatory response and how already compromised mechanisms of aging biology may modulate the severity and the consequences of such a response. Even more relevant is the rising evidence that immunobiography, the individual’s history of exposure to certain microorganisms (e.g. HIV and CMV) or antigens, may condition the degree and characteristics of a current inflammatory response to various stimuli [24, 32–34]. The underlying process by which the memory of previous exposures to inflammatory bouts modulates the innate response to a new antigen exposure is unknown but the existence of such memory has been demonstrated in epidemiological studies and is considered to be epigenetic in nature. In fact, immunobiography may be the mechanism by which obesity and the metabolic syndrome, both of which are characterized by chronic inflammation and abnormal production of cytokines (TNF, IL-1, and IL-6) and altered immune T cell response, appear to increase the risk of infection as well as its severity and consequences, particularly regarding COVID-19-related outcomes [35, 36].

In SARS-CoV-2-induced ARDS, the early stages are characterized by a massive production of inflammatory cytokines, formally referred to as the cytokine storm syndrome (CSS), which increases vascular permeability, vascular paralysis, hypovolemic shock and in its most overt form even multiorgan failure and death [37]. During the CSS, there is overproduction of IL-6, TNFα, and IL-1β, the main triad of inflammatory mediators, but there is also increased plasma levels of IL-2, IL-7, IL-10, granulocyte colony- stimulating factor (G- CSF), IP-10, MCP1, and macrophage inflammatory protein 1α (MIP1α) [10, 11] [37]. In hospitalized COVID-19 patients, IL-6 levels increase over time and its blood level is a robust risk factor for adverse outcomes, including in-hospital death [3, 7–9]. Interestingly, at least in studies conducted in adult and older COVID-19 patients, prognostic factors for mortality are the same as those found associated with a high likelihood and prospective risk of chronic inflammation, namely older age, male sex, obesity, smoking, cardiovascular diseases, asthma or other respiratory disease, cancer, autoimmune diseases, multimorbidity, and frailty [38]. Shared risk factors suggest that the same mechanisms underlying inflammaging are also those that modulate the clinical course of COVID-19 across a wide range of severity, from a mild flu-like syndrome to a severe respiratory failure with high mortality risk.

A puzzling possibility is that COVID-19 infection may predispose individuals to inflammaging through innate immunological memory even when the acute clinical syndrome is mild and clinically resolved in a few days with no apparent immediate consequences. Indeed, there is a strong rationale to support the hypothesis that acute SARS-Cov-2 infection induces subclinical damage accumulation that on the one hand predisposes the individual to a chronic pro-inflammatory state and on the other hand diminishes the capacity to fully benefit from a strong immune response in the context of an infection or a trauma. At the current stage, not enough data have been accumulated to address this question, but certainly as data accumulate more clarity will emerge on this issue. Here, we outline some of the hypothetical mechanisms that support this viewpoint and outline a future research agenda that can directly address this question by collecting longitudinal data in a cohort of patients who developed COVID-19 across a wide range of severity.

## Learning a lesson from the long-term sequalae of ARDS, SARS, cytokine storm syndrome: the post-ICU syndrome

There is evidence that severe infections are followed by long-term adverse sequelae including maintaining elevated levels of cytokines and having accelerated decreased functional capacities, similar to those seen in aging such as long-term physical, cognitive, and psychological impairment, even in patients that appear to have recovered from their initial insult such as loss of pulmonary function in acute respiratory disease syndrome (ARDS).

In a study by Herridge et al. [39], five years after leaving the ICU, patients who recovered from ARDS had long-term physical and psychological sequelae including exercise limitations and decreased physical quality of life. Despite normal to near normal pulmonary function, their 6-min walk distance was 436 m (76% of predicted distance) and the Physical Component Score on the Medical Outcomes Study 36-Item Short-Form Health Survey was 41 (the mean norm score matched for age and sex is 50). Having coexisting illness lowered the chance of being above 80% of the predicted distance by an OR of 0.48 per each additional illness. Younger patients did have a better recovery than older patients, but at 5 years, neither group returned to normal predicted levels of physical function. Fifty-one percent of patients reported having depression or anxiety. Patients with more coexisting illnesses incurred greater 5-year costs. The mortality rate of the individuals initially followed was 8% in the first year (median age of death 50 y/o), and 19% by year five. These data may suggest that the acute event that required admission to the ICU caused a process that lead to accelerated frailty, which is mainly suggested by the progressive decline of mobility, one of the main and early characteristics of frailty. However, the mechanism by which such an acute event triggers a long-term process of functional decline remains unclear. A possible hypothesis, outlined more in details later in this article, is that the acute insult causes an overt destabilization of the basic cellular homeostatic mechanisms and the resulting cellular stress leads to the accumulation of senescent cells in multiple tissue. Even after the acute condition is resolved, the residual burden of senescent cells may cause chronic inflammation through the continuous production of SASP proteins, with all the consequences outlined above.

In the context of ARDS, CSS may be viewed as the critical event that starts this process. CSS has also been observed in SARS and Middle East Respiratory Syndrome (MERS), both also caused by coronaviruses, and results in high levels of pro-inflammatory cytokines similar to COVID-19. In SARS, patients with severe disease had high levels of serum pro-inflammatory cytokines (IFN-γ, IL-1, IL-6, IL-12, and TGFβ) and chemokines (CCL2, CXCL10, CXCL9, and IL-8) compared to patients with mild disease [40]. In MERS, patients with severe disease had high levels of serum pro-inflammatory cytokines (IL-6 and IFN-α) and chemokines (IL-8, CXCL- 10, and CCL5) compared to mild or moderate disease [41]. The presence of a pro-inflammatory background that is typical of inflammaging likely exacerbates the severity of CSS. For example, in a mouse model, the presence of inflammaging increased the incidence and lethality of the cytokine storm following inducement with systemic immunotherapy [42].

Many ICU patients that experience severe and prolonged illness suffer from post-intensive care syndrome (PICS) that manifests as cognitive, psychological and physical disabilities and a large percentage of them never fully recover their well-being and functional status [43]. Physical impairment in PICS often involves muscle weakness, extreme fatiguability, mood disorders, polyneuropathy, and symptoms of deconditioning that only mildly respond to exercise programs [44]. Interestingly, these clinical features are similar to those experienced by older individuals with inflammaging and frailty-related sarcopenia [34, 45]. Indeed, survivors of SARS, ARDS, CSS, and PICS, all elicit physical and cognitive deficits that could be characterized as accelerated inflammaging raising the possibility that COVID-19 survivors may face accelerated inflammaging as well, with substantial long-term consequences on their well-being. For the COVID-19 experience, the long-term effects on health and functional status may be further worsened by the implementation of social distancing, socioeconomic stress, and isolation that is unprecedented in world history, and whose long-term consequences on individuals and society will be the subjectof intensive research in the years to come.

## COVID-19 and mechanisms of inflammaging in older individuals

**Fig. 2:**
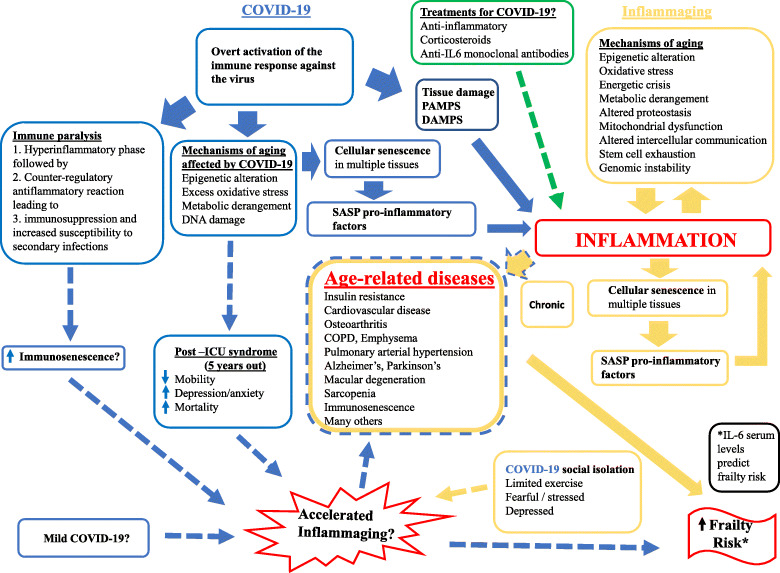
COVID-19 and mechanisms of inflammaging in older individuals. *Inflammaging (highlighted in yellow)*. Dysfunction of the basic mechanistic “hallmarks” and/or “pillars” of aging, including epigenetic alteration, oxidative stress, metabolic derangement, and genomic instability among other mechanisms, results in inflammation. Aging is associated with cellular senescence in many tissues, and it has been hypothesized that “in vivo,” similar to what has been demonstrated “in vitro,” such permanent state of proliferative arrest can be induced by many different cellular stressors in humans. One characteristic of cellular senescence is the secretion of a number of very active compounds (senescence associated secretory phenotype, or SASP) that include pro-inflammatory cytokines, which may leak in the circulation and contribute to a cycle of chronic inflammation with age (inflammaging). The accumulation of senescent cells in various tissues with age, especially with concurrent obesity, is considered part of the mechanism by which age is the strongest risk factor for many chronic diseases including atherosclerosis, osteoarthritis, chronic obstructive pulmonary disease (COPD), sarcopenia, and immunosenescence. These age-related diseases lead to an increased risk of morbidity, disability, and frailty. Serum levels of IL-6, a pro-inflammatory cytokine and a strong biomarker of inflammaging, also predict incident disability and frailty. *COVID-19 (highlighted in blue)*. COVID-19 infection may hypothetically negatively impact several hallmarks of aging and theoretically accelerate inflammaging with an increased risk of age-related diseases (*dashed blue lines*) through various interrelated mechanisms. The overt activation of the immune response against the virus through the release of PAMPs and DAMPs can activate inflammation, which can adversely impact mechanisms of aging through the production of excessive oxidative stress, metabolic derangement, and DNA damage that can trigger cellular senescence and the production of SASP pro-inflammatory factors in multiple tissues. The immune response against the virus and the frequently superimposed bacterial infections may also represent an overwhelming challenge to the immune system and exhaust or dysregulate some of the physiological system, directly contributing to immunosenescence. Immunosenescence affects different aspects of the immune system that have vital implications to developing resistance to SARS-CoV-2 infection but may also contribute to deleterious sequalae, such as the maintenance of chronic inflammation. It is also possible that SARS-CoV-2 activates inflammation so strongly that there could be an exhaustion of the capacity to produce an inflammatory reaction (immune paralysis) such as seen in conditions like sepsis. The initial hyperinflammatory (cytokine storm syndrome; CSS) phase is followed by a powerful counter-regulatory, anti-inflammatory reaction and though the parameters of inflammation are still high, there is a reduced capacity to react to a new inflammatory insult, which can lead to immunosuppression and increased susceptibility to secondary infections. Many ICU patients that experience severe and prolonged illness suffer from post-intensive care syndrome (PICS) that manifests as cognitive, psychological and physical disabilities and a large percentage of them never fully recover their well-being and functional status. These clinical features are similar to those experienced by older persons with inflammaging and other age-related diseases such as sarcopenia, who are at increased risk for frailty. Survivors of SARS, ARDS, CSS, and PICS, all elicit physical and cognitive deficits that could be characterized as accelerated inflammaging raising the possibility that COVID-19 survivors may face accelerated inflammaging as well, with substantial long-term consequences on their well-being. For the COVID-19 experience, the ramifications of this syndrome may be worsened by the implementation of social distancing, socioeconomic stress and isolation that is unprecedented in world’s history, and whose long-term consequences on individuals may include accelerated inflammaging and development of age-related diseases and increased frailty risk. Whether blunting the cytokine storm by any type of intervention (*highlighted in green*) may prevent these long-term consequences, such as the development of inflammaging, is unknown and is one of the fundamental questions that should be addressed in future surveillance studies of COVID-19 patients, particularly older individuals, who recover from the acute infection and even those who face unprecedented COVID-19 social isolation who may have reduced physical activity and increased fear, stress, and depression

In addition to SARS-CoV-2 affecting older individuals more severely in the short-term, there is some initial evidence that COVID-19 may have greater long-term consequences in older people through biological processes set in motion after the acute infection has subsided sustaining inflammaging **(**Fig. 2). We have previously proposed that the dysfunction of the basic mechanistic “hallmarks” [46] and/or “pillars” [23] of aging, including adaptation to stress, genomic instability, mitochondrial dysfunction, epigenetic alteration, macromolecular damage, metabolism/dysregulated nutrient sensing, altered intercellular communication, loss of proteostasis, stem cell exhaustion, cellular senescence, and regeneration, results in inflammation [24, 47]. There is rationale to hypothesize that COVID-19 infection may negatively impact several hallmarks of aging, namely: 1) As we already mentioned, COVID-19 infection produces excess oxidative stress, metabolic derangement, and DNA damage that may trigger cellular senescence in multiple tissues; 2) The viral infection directly causes severe tissue damage with release of PAMPs and DAMPs, which activate inflammation; 3) The overt activation of the immune response against the virus and the frequently superimposed bacterial infections may represent an overwhelming challenge to the immune system and exhaust or dysregulate some of the physiological system, directly contributing to immunosenescence. Part of such dysregulation may result from epigenetic changes aimed at modulating gene expression to optimally respond to incoming infections, a sort of epigenetic adaptation to repeated changes from the environment. In the following part of this manuscript, we discuss each one of these hypothetical mechanisms.

Cellular senescence is a permanent state of proliferative arrest that can be induced by many different cellular stressors. The arrest of replication is currently interpreted as stress-response mechanisms in the presence of homeostatic instability that may lead to neoplastic transformation. Other main characteristics of cellular senescence are the secretion of a number of very active compounds (SASP) that include pro-inflammatory cytokines, chemokines, growth factors, proteases, insoluble proteins/extracellular matrix components and other protein and non-proteins signaling molecules, with a list that is still growing as new research is published. Cellular senescence has been implicated in many chronic diseases including atherosclerosis, cancer, and osteoarthritis and is hence an emerging target for the development of new treatments [48]. In fact, the accumulation of senescent cells in various tissues with age, especially with concurrent obesity, is considered part of the mechanism by which age is the strongest risk factors for many chronic diseases [49]. There is evidence that viral infections may cause cellular senescence as a host cells’ response to fighting of viruses that endanger genomic integrity [50]. For instance, vesicular stomatitis virus replication is markedly impaired in both primary and tumor senescent cells in comparison with non-senescent cells [51]. Other examples of viral induced cellular senescence include dengue, Epstein-Barr, HIV-1, measles, as well as the virulence factor, NSI protein, of the influenza A virus [52]. While it is still unclear whether SARS-CoV-2 induces cellular senescence, this possibility should be explored in the future in older COVID-19 survivors.

PAMPs and DAMPs interact with PPR sensors expressed on the cell surface and cytoplasm of many immune cell types thereby triggering inflammatory responses that contribute to inflammaging. Lung and upper respiratory tissue pathology studies have shown that SARS-CoV-2 infection results in severe local tissue damage [53, 54]. Fragmentation of cells releases PAMPs and DAMPs and activates inflammation through local cells including, in the case of the lung, alveolar epithelial cells and alveolar macrophages that release pro-inflammatory cytokines and chemokines that attract immune cells including monocytes and T cells [10]. In SARS-CoV-2, similar to SARS-CoV-1 and MERS-CoV, adaptive mechanisms evade innate sensing triggered by engagement with PPR, and consequential cytokine secretion and IFN signal transduction. These mechanisms of evasion include shielding dsRNA with membrane-bound compartments, guanosine capping and methylation of CoVs non-structural proteins, and encoding NSP15, an endoribonuclease that cleaves 5′ polyuridines that are formed during viral replication, which would be detected by MDA5 in the cytosol [55]. Despite these defenses to evade innate sensing, the SARS-CoV-2 induced cytokine storm is still likely initiated by a combination of viral PAMPs and host DAMPs [55].

Direct attempts to block the pro-inflammatory cytokines released during the SARS-CoV-2 induced CSS are being examined since it has been shown that its severity correlates with COVID-19 morbidity and mortality. In particular, monoclonal antibodies against the IL-6 signaling pathway have been proposed [56]. The anti-IL-6R antibodies tocilizumab and sarilumab and the anti-IL-6 antibody siltuximab are currently being tested in 13 clinical trials for efficacy in managing COVID-19 CSS and pneumonia [55]. While some if these studies found a positive response in some biomarker and clinical values, it is still too soon to say whether they substantially affect clinical outcomes and, in particular, whether they reduce the risk of possible long-term sequalae of inflammaging in ICU patients.

During the aging process, the immune system appears to maintain a permanent state of mild activation and when stimulated, its dynamic response is compressed. The combination of a chronic pro-inflammatory state, as seen in inflammaging, and a concurrent reduced ability to mount an effective defense is often referred to as immunosenescence [57]. Immunosenescence affects different aspects of the immune system that have vital implications to developing resistance to SARS-CoV-2 infection but may also contribute to deleterious sequalae, such as the maintenance of chronic inflammation. In older individuals, both T and B cell proliferation are reduced after activation, with consequential reduction in the number of effector cells, which reduces the ability to amount efficacious immune responses to new threats [58]. The unopposed viral load may be a major determinant of the cytokine storm, although with a mechanism that is not clear, and the damage induced by cytokines drives the severity of the syndrome and its clinical consequences. However, whether blunting the cytokine storm by any type of intervention may prevent long-term consequences, such as the development of inflammaging is unknown and is one of the fundamental questions that should be addressed in future surveillance studies of COVID-19 patients who recover from the acute infection.

A final hypothesis is that SARS-CoV-2 activates inflammation so strongly that there could be an exhaustion of the capacity to produce an inflammatory reaction (immune paralysis). In individuals with immune paralysis, though the parameters of inflammation are still high, there is a reduced capacity to react to a new inflammatory insult. This condition is often observed in severe septic shock characterized by high fever, pathologically low blood pressure, organ hypoperfusion and respiratory failure due to an uncontrolled pro-inflammatory response termed systemic inflammatory response syndrome [59, 60]. The hypothetical mechanism underlying this severe condition is that PAMPs from bacterial, fungal, and viral organisms and DAMPs from host injury bind to PRR on innate immune cells, which results in the production of pro- and anti-inflammatory cytokines (cytokine storm) to produce many cellular and counter responses including enhanced phagocytic activity, vascular endothelial injury/capillary leak, synthesis in the liver of acute phase proteins, leukocyte chemotaxis to the site of inflammation/infection, and activation of the coagulation system [59]. The initial hyperinflammatory (cytokine storm) phase of sepsis is followed by a powerful counter-regulatory, anti-inflammatory reaction (originally called compensatory anti-inflammatory response syndrome, now immune-paralysis) [59, 61], characterized by apoptosis of immune effector cell and cellular (especially T cell) exhaustion leading to immunosuppression and increased susceptibility to secondary infections. It has been observed that in some cases, SARS-CoV-2 elicits a reaction similar to CSS seen in sepsis. A puzzling hypothesis is that after the resolution of immune-paralysis, a dysfunctional immune system becomes chronically upregulated (ergo the high levels of inflammatory markers) to deal with small daily challenges that in normal individuals would barely trigger an inflammatory reaction, and that such chronic upregulation ultimately sustains inflammaging. The mechanism for the upregulation is unknown but, ultimately, long-term adaptation is only possible by a different setting in the regulation of gene expression, which can only happen through epigenetic tuning. Thus, it is possible that a changed chromatin state occurs characterized by the incorporation of new histone variants, DNA methylation patterns, and histone modification patterns that result in different transcriptional programs activated by transcription factors, resulting in the recruitment of different chromatin modifiers [62]. Epigenetic regulators that circulate in the blood, such as MiRNAs, appear to change with age [63] and several have been linked to inflammation (inflamma-miRs), including miR-21, − 126 and -146a that target the NF-κB pathway [64]. In defending against SARS-CoV-2 infection, there may be beneficial host epigenetic changes that upregulate the proinflammatory innate immune response [65], an adaptive response that may be useful in the immediate term but harmful in the long term. In addition, host innate immune system epigenetic changes induced by SARS-CoV-2 to evade host defenses can also lead to a changed chromatin state. For example, in SARS-CoV-1 infection specific changes of histone methylation delay the expression of host interferon-stimulated genes therefore allowing for viral replication, while repressive histone modifications with MERS-CoV downregulate expression of interferon-stimulated gene subsets [66]. In MERS-CoV, epigenetic changes cause a global downregulation of reactive mechanisms to antigen-presentation gene expression [67]. It is reasonable that similar epigenetic changes occur in SARS-COV-2.

Regarding possible COVID-19 treatments that intersect with inflammaging, intriguing results from the RECOVERY (Randomised Evaluation of COVid-19 thERapY) randomized clinical trial of over 11,500 patients in the UK reported that low-dose dexamethasone (6 mg once per day for up to ten days, either by mouth or by intravenous injection) in ~ 2100 patients reduced mortality by 29% in ventilated patients and by 11% in other patients receiving oxygen only, with no benefit for patients who did not require respiratory support [68]. Corticosteroids could be used to dampen CSS, though their usage did not show previous survival benefit with MERS and ARDS, likely because corticosteroids delayed the clearance of the virus [69]. In COVID-19 and other CSS, the benefits of corticosteroids may be dependent on the time of administration. The effect is probably maximum before the full development of the cytokine storm [70]. Treatment of inflammaging for age-related diseases may also need to balance dosage and timing of anti-inflammatory therapeutics, including steroids or other immune modulating modalities to most benefit patients.

## The follow-up of COVID-19 older patients: a public health strategy

Based on the review of the literature, we predict that older persons who recovered from COVID-19 will be more likely to develop inflammation and its consequences in terms of excess of morbidity, disability, and frailty. Also, these individuals are more likely to experience accelerated immunosenescence and therefore will be more susceptible to infections. If this hypothesis is true, it would be important to set up a longitudinal follow-up of COVID-19 patients in order to identify those subjects at higher risk of developing age-related diseases and be prepared for early interventions that may reduce the long-term effects of acute COVID-19. This wide research agenda may involve several important actions:

1. Create A NATIONWIDE REGISTRY of older patients (> 60 years) who survived a COVID-19 infection across a wide range of severity. In addition to basic demographics, the registry should collect information on 1) The severity and the clinical course of the acute event and short- and long-term complications; 2) Extensive information on biological, physiological and functional biomarkers of aging, including both physical and cognitive aspects; 3) Standard measure of prevalent chronic diseases, including a validated measure of multimorbidity; 4) information on risk factors including environmental behavioral and social risk factors.
2. The registry should be complemented by a NATIONWIDE BIOBANK including biological specimens (blood derivatives but also other biofluids) for the subjects included in the registry.
3. The registry and the biobank should be the basis for a NATIONWIDE LONGITUDINAL FOLLOW-UP of patients who survived COVID-19 infection. The follow-up should collect information of the long-term respiratory, neurological and metabolic pathological outcomes, as well as physical and cognitive function. Analysis of this database should allow a better identification of the individuals characteristics associated with adverse health outcomes.

Using this set of data, it should also be possible to test the hypothesis that COVID-19 infection, similar to other types of severe infection, leads to accelerated biological aging and its consequences.

## Conclusions

COVID-19 changed the entire world; life as we have experienced it in the past may never come back. Individuals and entire societies have shown incredible and unexpected resilience in adapting to this challenge by implementing dramatic changes of unprecedented magnitude. The pandemic expansion of the virus affected millions of individuals and caused substantial morbidity and mortality that is still challenging the stability of the health care system of whole countries. Older people, and especially those affected by multimorbidity and frailty as well as those living in long-term care facilities have paid the steepest price. Even after the pandemic ends, the possibility exists that morbidity from COVID-19 will not fully disappear. The challenges induced by the pandemic also created a natural experiment that could be exploited to better understand aging and immunosenescence. Future follow-up studies can tell us whether the SARS-CoV-2 infection affects the trajectories of health and physical/cognitive function with aging and whether specific biological and clinical characteristics of the acute phase further influence these trajectories. Similarly interesting will be to follow older individuals who were not affected by the pandemic during the same period but underwent social distancing and had consequential changes in routines for physical activity, social interaction, nutrition, health care and other basic aspects of life for a relatively long period and because of these challenges may develop long-term effects.

## Data Availability

Not applicable.
